# Anti-mullerian hormon level and polycystic ovarian syndrome diagnosis

**Published:** 2015-04

**Authors:** Shahrzad Zadehmodarres, Zahra Heidar, Zahra Razzaghi, Leili Ebrahimi, Kaveh Soltanzadeh, Farhang Abed

**Affiliations:** 1 *Infertility and Reproductive Health Research Center, Shahid Beheshti University of Medical Sciences, Tehran, Iran *; 2 *Laser Application in Medical SciencesResearch Center, Shahid Beheshti University of Medical Sciences, Tehran,Iran.*; 3 *Infertility Ward, Mahdieh Hospital, Shahid Beheshti University of Medical Sciences, Tehran, Iran.*

**Keywords:** *Anti-mullerian hormon*, *Polycystic ovarian syndrome*, *Hirsutism*, *Rotterdam criteria*

## Abstract

**Background:**

Polycystic ovarian syndrome (PCOS) is a common endocrinopathy that accompanied with long term complications. The early diagnosis of this syndrome can prevent it.

**Objective:**

The aim was to determine the role of anti-mullerian hormon (AMH) in PCOS diagnosis and to find cut off level of it.

**Materials and Methods:**

In this cross sectional study, 117 women between 20-40 years old were participated in two groups: 60 PCOS women (based on Rotterdam criteria consensus) as the case group and 57 normal ovulatory women as the control group. In day 2-4 of cycle, transvaginal sonography was performed and serum hormonal level of AMH, luteinizing hormone (LH), follicle stimulating hormone (FSH), estradiol (E_2_), testosterone, fasting blood sugar (FBS), thyroid stimulating hormone (TSH), and prolactin (PRL) were measured in all of participants. For all of them score of hirsutism (base on Freeman-Galloway scoring) was determined.

**Results:**

There were statistically significant in irregular pattern of menstruation, AMH and FSH level, and presence of hirsutism between two groups. But regarding mean of age, body mass index, plasma level of PRL, TSH, LH, Testosterone, FBS, and E_2_ differences were not significant. Construction by ROC curve present 3.15 ng/ml as AMH cut off with 70.37% sensitivity and 77.36% specificity in order to PCOS diagnosis.

**Conclusion:**

AMH with cut off level of 3.15 ng/ml with sensitivity 70.37% and specificity 77.36% could use for early diagnosis of PCOS patients.

## Introduction

Polycystic ovarian syndrome (PCOS), a common endocrinopathy characterized by oligo-or anovulation, clinical or biochemical hyperandrogenmia, and polycystic ovaries on ultrasonography, affects 5-10% of women of reproductive age (1,2). Recent studies have shown that 50% of women with PCOS fulfill the criteria of metabolic syndrome and that PCOS is frequently associated with insulin resistance accompanied by compensatory hyperinsulinemia, resulting in an increased risk for the development of type 2 diabetes mellitus and cardiovascular disease (2,3). In comparison with healthy women, PCOS have higher level of anti-mullerian hormone (AMH) that is a peptide produced by the granulosa cells of follicles that is widely considered as a highly sensitive marker of ovarian reserve (3). Previous studies have suggested that AMH may play a pathogenetic role in follicular status of PCOS (4,5). Because of long term effect of PCOS and metabolic syndrome, early diagnosis of this endocrinopathy is very important. Our purpose in this study was to find any relation between AMH levels and PCOS diagnosis.

## Materials and methods

In this cross sectional study, 117 women between 20-40 years old referred to Infertility clinic, Mahdieh Hospital, Tehran, Iran from 2012 to 2013 were participated in two groups. Written informed consent was obtained from all of them. The case group consisted of 60 PCOS women (based on Rotterdam criteria consensus) and the control group was 57 women with normal ovulatory state. Our study protocol was approved by Ethics Committee of Mahdieh Hospital.

Inclusion criteria in case group was PCOS diagnosis, 20<age<40, and presence of both ovaries. PCOS was ascertained, using the Rotterdam consensus statements, as the presence of two of the following three criteria: PCO morphology (more than 12 follicle with size 2-9 mm or ovarian volume more than 10 ml in one ovary), clinical or biochemical hyperandrogenism (hirsutism with score ≥8 based on Freeman-Galloway scoring or testosterone >2.5 nmol/l, free testosterone ≥0.6 nmol/l), and oligomenorrhea (cycle length >35 days). Exclusion criteria were history of ovarian surgery, and induction ovulation in recent 6 month. Thyroid and adrenal function tests were normal in both groups and they did not use OCP in last month.

All of participants were examined carefully at beginning and their demographic data such as age, gravity, weight, height, waist, hip circumference, and history of medical state were written in information sheet. In day 2-4 of cycle, transvaginal sonography (Honda, Japan) was performed and serum hormonal level of AMH (ELIZA, Beckman-culter, ng/ml,), luteinizing hormone (LH) (RIA, mIU/ml), follicle stimulating hormone (FSH), (RIA, mIU/ml), estradiol (E_2_) (ECL, pmol/ml), testosterone (ECL ng/ml), fasting blood sugar (FBS) (mg/dl), thyroid-stimulating hormone (TSH) (mIu/l), and prolactin (PRL) (ng/ml) were measured.

**Statistical analysis**

Statistical analysis was performed with statistical package for the social science (SPSS Inc, Chicago, Illinois, USA) version 16.0. The data were analyzed by using the Chi-square, fisher exact test, and Student’s *t*-test. A p<0.05 was considered as significant.

## Results

Our results show that the case and control groups were matched respecting the age and BMI. Mean of AMH level in case group was 7.14±6.53ng/ml and in controls was 3.34±3.45 ng/ml which the difference was statistically significant (p=0.001). Also, FSH level was significantly difference in two groups (p=0.0001) (Table I). PCO morphology in none of controls was seen. Overall 29 (48.3%) cases and 11 (19.9%) controls have hirsutism with score >8 (p=0.001). Irregular mense in 37 (61.7%) cases and 15 (26.3%) controls were seen (p=0.001). In four (6.7%) cases and one of the controls (1.8%) hyperprolactinemia was seen (p=0.89).

**Figure 1:**
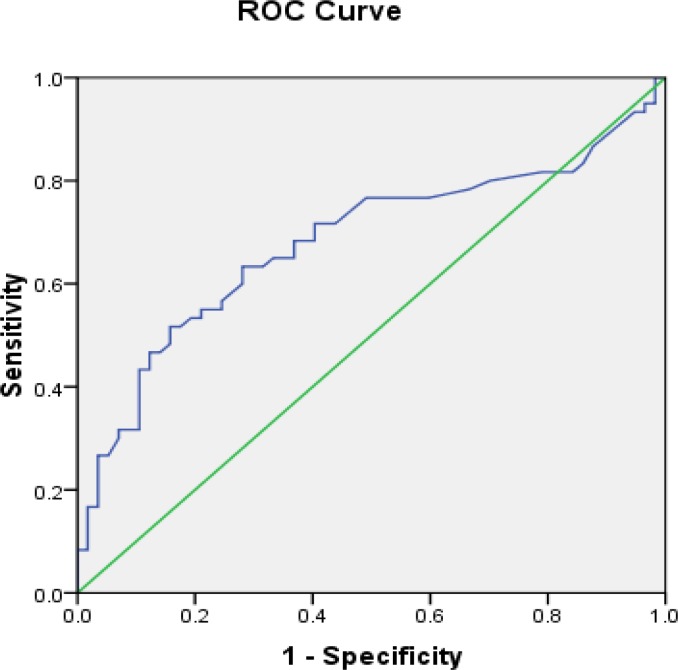
Roc curve of AMH (blue line) with reference line (green line).

Differences in plasma level of LH, TSH, FBS and E_2_ were not statistically significant (Table I). To determine of AMH diagnostic cut off, ROC curves were constructed that presented 3.15 with 70.37% sensitivity and 77.36% specificity and positive predictive value (PPV)=76% and NPV(negative predictive value)=71.93 in order to determinate AMH cut off level in diagnosis of PCOS (Figure 1).

**Table I:** Demographic characteristics of case (PCOS women) and control groups

**Variables**	**Case group**	**Control group**	**p** **-** **value** *
Age (Year)	27.07 ± 4.49	28.68 ± 4.98	0.072
BMI (Kg/m^2^)	29.02 ± 6.53	28.76 ± 3.41	0.389
AMH (ng/ml)	7.14 ± 6.53	3.34 ± 3.45	0.001
FSH (mIu/ml)	4.53 ± 1.62	6.88 ± 5.56	0.0001
LH (mIu/ml)	5.96 ± 2.93	5.58 ± 3.09	0.452
TSH(mIU/ml)	3.03 ±6.27	2.73± 2.16	0.34
FBS )mg/dl)	92.81±11.14	93.74±10.11	0.755
Testosterone nmol/l (	0.52±0.63	0.52±0.28	0.324
Estradiol (pg/ml(	65.42±22.84	77.58 ±60.80	0.608

Data are presented as mean±SD.

BMI: body mass index; AMH: anti-mullerian hormone; FSH: follicle stimulating hormone; LH: luteinizing hormone, TSH: thyroid-stimulating hormone, FBS: fasting blood sugar

* Student’s* t* test

## Discussion

As mentioned earlier because of long term sequel of PCOS including infertility, endometrial hyperplasia, metabolic syndrome, and cardiovascular risk factor, early identification of at risk women would be very useful. Once the diagnosis of PCOS is made, additional evaluation is suggested including a cardio metabolic risk assessment, as well as screening for mood disorder and sleep apnea, screening for diabetes mellitus and for women pursuing fertility assessment of ovulatory status (6). The present study demonstrated that there is positive correlation between AMH level and PCOS diagnosis and 3.15 nmol/ml as cut off level (with sensitivity and specificity of 70.37% and 77.36% respectively) could use for PCOS diagnosis (p=0.001). In other researches AMH>7.7 ng/ml (3) and >3.5 ng/ml (9) represented as diagnostic cutoff level for PCOS.

The differences of FSH, PCOS morphology, hirsutism and irregular mense between patients and normal women were statistically significant but testosterone, PRL, FBS, E_2_ and BMI differences between case and control groups were not significant. In another study there was correlation between PCO morphology and AMH level in regular cycle adolescent but in our research and Eilertsen*,* PCOS morphology between case and controls was significant different (7,8). There is some debate about value of sonographic finding in PCOS diagnosis Vise versa our finding, in Villarroel study PCOM was a common finding in normal ovulatory women(8) thus there is need to more research in this field.

In two studies, androgen level in PCOS patients was significantly higher, and in one study androgen level in overweigh (BMI >27) was higher but our study did not show this different race in study groups may be responsible (11-13). In some studies there was linear relation between AMH and testosterone level, hirsutism and oligomenorrhea but our study did not find such result (7, 11, 12). It needs to mention that our population study group selected from infertile women and it maybe affected our result. This is obvious that larger studies in different rational group of patients are needed to determine the accurate diagnostic cut off level of AMH.

## Note

***This article extracted from MD thesis (Dr. Ebrahimi).***
